# Performance of Fluxgate Magnetometer with Cu-Doped CoFeSiB Amorphous Microwire Core

**DOI:** 10.3390/s24010309

**Published:** 2024-01-04

**Authors:** Bin Wang, Weizhi Xu, Xiaoping Zheng, Sida Jiang, Zhong Yi, Peng Wang, Xiaojin Tang

**Affiliations:** 1 Department of Automation, Tsinghua University, Beijing 100084, China; wangbin20@mails.tsinghua.edu.cn; 2 Department of Space Magnetism and Application Research, Beijing Institute of Satellite Environmental Engineering, Beijing 100094, China; yizhong6808@sina.com (Z.Y.); aiveidy@126.com (X.T.); 3 National Key Laboratory of Space Environment and Matter Behaviors, Harbin Institute of Technology, Harbin 150001, China; 23s109222@stu.hit.edu.cn (W.X.); 21b309024@stu.hit.edu.cn (P.W.)

**Keywords:** CoFeSiB microwire, Cu doping, nanocrystalline structures, magnetic fluxgate sensor

## Abstract

In this study, we investigated the effects of Cu doping on the performance of CoFeSiB amorphous microwires as the core of a fluxgate magnetometer. The noise performance of fluxgate sensors primarily depends on the crystal structure of constituent materials. CoFeSiB amorphous microwires with varying Cu doping ratios were prepared using melt-extraction technology. The microstructure of microwire configurations was observed using transmission electron microscopy, and the growth of nanocrystalline was examined. Additionally, the magnetic performance of the microwire and the noise of the magnetic fluxgate sensors were tested to establish the relationship between Cu-doped CoFeSiB amorphous wires and sensor noise performance. The results indicated that Cu doping triggers a positive mixing enthalpy and the reduced difference in the atomic radius that enhances the degree of nanocrystalline formation within the system; differential scanning calorimetry analysis indicates that this is due to Cu doping reducing the glass formation capacity of the system. In addition, Cu doping affects the soft magnetic properties of amorphous microwires, with 1% low-doping samples exhibiting better soft magnetic properties. This phenomenon is likely the result of the interaction between nanocrystalline organization and magnetic domains. Furthermore, a Cu doping ratio of 1% yields the best noise performance, aligning with the trend observed in the material’s magnetic properties. Therefore, to reduce the noise of the CoFeSiB amorphous wire sensor, the primary goal should be to reduce microscopic defects in amorphous alloys and enhance soft magnetic properties. Cu doping is a superior preparation method which facilitates control over preparation conditions, ensuring the formation of stable amorphous wires with consistent performance.

## 1. Introduction

The performance of fluxgate sensors relies heavily on the characteristics of the magnetic core material, which, in turn, are influenced by its internal microstructure. Amorphous alloy cores, lacking an internal lattice structure, are highly desirable owing to their excellent soft magnetic characteristics and minimal noise. Notably, CoFeSiB amorphous microwires cores have reduced the noise of fundamental mode orthogonal fluxgates (FM-OFG) to an impressive 750 fT/$\sqrt{Hz}$ [1], matching the noise level of superconducting quantum interference devices (SQUIDs). This advancement highlights the vast potential of fluxgate sensors in various fields, including magnetic particle detection [2], biological magnetic field detection [3], and space field detection [4].

The practical application of fluxgate sensors is limited because of the instability of amorphous materials. Commercial CoFeSiB amorphous microwires of the same model from a single company exhibit vastly different noise performances [5], hindering large-scale manufacturing and deployment. To overcome this challenge, a correlation between the microstructure of amorphous microwires and noise performance must be established while carefully analyzing the micro-mechanism underlying the performance disparities between CoFeSiB amorphous microwires. This understanding will pave the way for enhancing the noise performance and stability of fundamental mode orthogonal fluxgate sensors.

Elemental doping has emerged as a vital technique for adjusting the microcosmic crystal structure of amorphous alloys [6]. Low Cu doping, in particular, can significantly improve the soft magnetic properties of certain amorphous alloys [7,8]. For CoFeSiB amorphous alloy, the near-insolubility of Cu in Co and Fe alloys at room temperature leads to the nucleation and refinement of nanocrystalline structures within alloys. Furthermore, α-(Co, Fe) nanophases can enhance the soft magnetic properties of the amorphous alloy [9], directly affecting the performance of amorphous magnetic cores. Moreover, elemental doping can modulate the glass state formation conditions of the CoFeSiB alloy [10], thereby reducing its noise [11], a potential determinant [12] of the noise performance of amorphous microwires used as fluxgate cores [13].

In this study, we investigated CoFeSiB amorphous microwires prepared using melt-extraction technology (MET). By doping with Cu, the positive enthalpy of mixing and reduced atomic radius differences due to Cu substitution weakened the glass-forming ability, and the microscopic crystal structure of CoFeSiB amorphous microwires was regulated, resulting in nanocrystalline phases on the amorphous substrate. Subsequently, amorphous microwires with varying doping ratios were transformed into FM-OFG sensors, and their noise performances were thoroughly tested at 1 Hz. Finally, we established the corresponding relationship between the microcosmic structure of the amorphous cores and the noise performance of the sensors, and the micro-mechanism underlying the regulation of amorphous alloys through element doping was investigated.

## 2. Preparation and Microstructure Characterization

**Figure 1:**
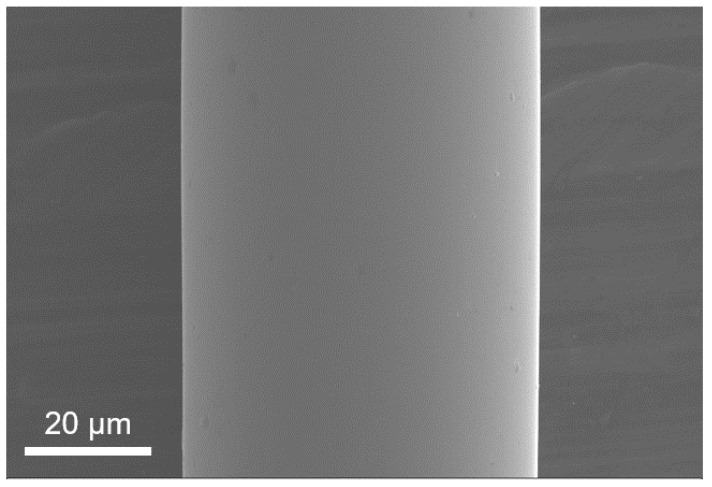
Microscopic morphology of Cu-doped amorphous microfilaments prepared using MET.

CoFeSiB amorphous microwires were primarily prepared using the glass coating method as the magnetic fluxgate core. However, an interaction exists between the glass coating and the wire core [14]. To avoid potential interference, this study employed melt-extraction technology (MET) to prepare CoFeSiB amorphous microwires with a high degree of amorphousness and without being influenced by the glass coating layer, which is highly suitable for microstructure analysis. The corresponding composition of the mother ingots was fabricated by arc-melting pure Co (99.99%), Fe (99.99%), Si (99.999%), B (99.7%), and Cu (99.99%) elements in an argon atmosphere and pouring into a 10 mm diameter copper mold for further extraction processing. During preparation, a parent phase alloy rod was melted in a vacuum crucible, and a certain amount of molten alloy was extracted through the wedge edge of a copper wheel, leading to rapid cooling and the formation of amorphous microwires. The appearance of a Co-based amorphous microfilament is given in Figure 1. The diameter of the amorphous microfilament is about 45 μm, the size is uniform, there are no defects such as grooves and sharp waves on the surface, and the degree of roundness is high, which indicates that high-quality Co-based amorphous microfilaments can be prepared using MET.

CoFeSiB amorphous wires were prepared with a basic nominal composition of Co_68.15_Fe_4.35_Si_12.25_B_15.25_. Cu was introduced to substitute B with doping ratios of 1% and 3%, resulting in samples labeled as Cu1 and Cu3, respectively. The specific nominal component proportions are provided in Table 1.

**Figure 2:**
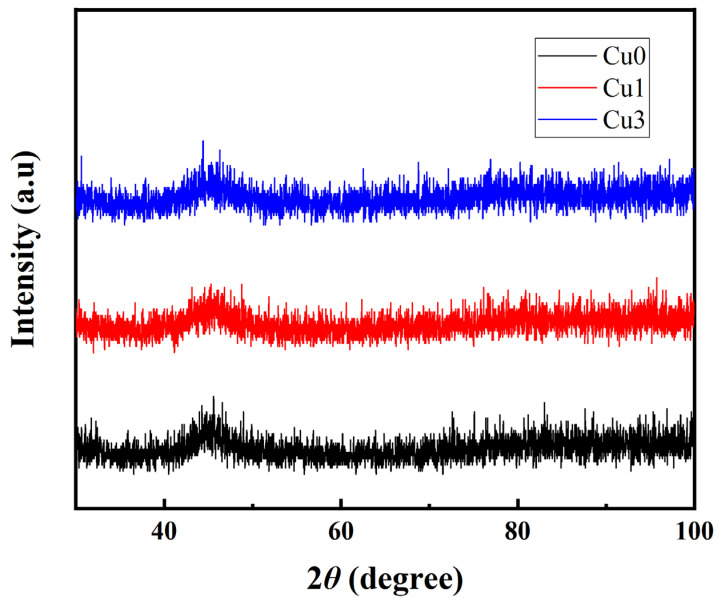
XRD patterns of amorphous microwires with Cu-doped amounts of 0%, 1%, and 3%.

Figure 2 presents the X-ray diffraction (XRD) patterns of the microwire samples. The absence of clear crystalline diffraction peaks and the presence of a wide diffuse peak indicates that the wire samples were predominantly in an amorphous state. When the amount of Cu doping is 0%, the diffraction peak strength of the filament is weak, indicating that the amorphization degree of the prepared microfilament is very high. When Cu is used to replace the B element in the Co-Fe-Si-B filament, the intensity of the diffuse scattering peak begins to increase. When the doping of the Cu element increases to 3%, the diffraction peak strength is the largest.

Transmission electron microscopy (TEM) was employed to examine the microstructure of amorphous microwires. The samples were evenly pasted to conductive rings at specific intervals and thinned to a thickness of several atomic layers using an ion thinning device.

**Figure 3:**
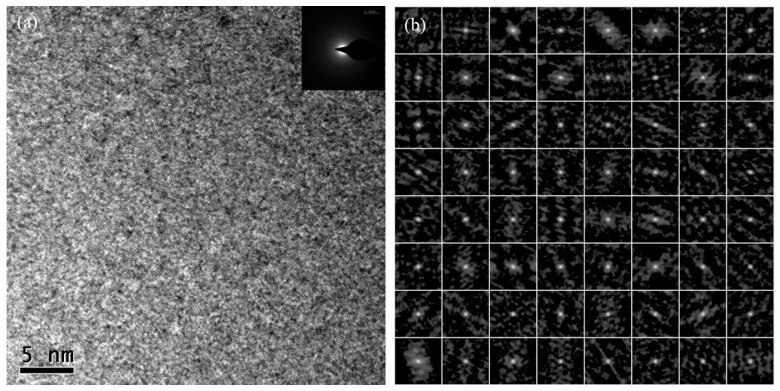
(**a**) HRTEM and (**b**) SAED images of the Cu0 sample.

**Figure 4:**
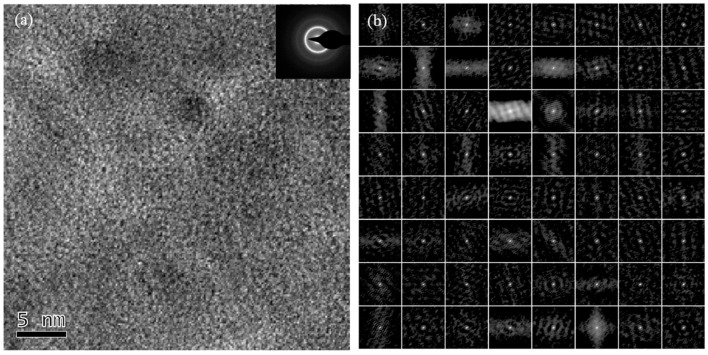
(**a**) HRTEM and (**b**) SAED images of the Cu1 sample.

**Figure 5:**
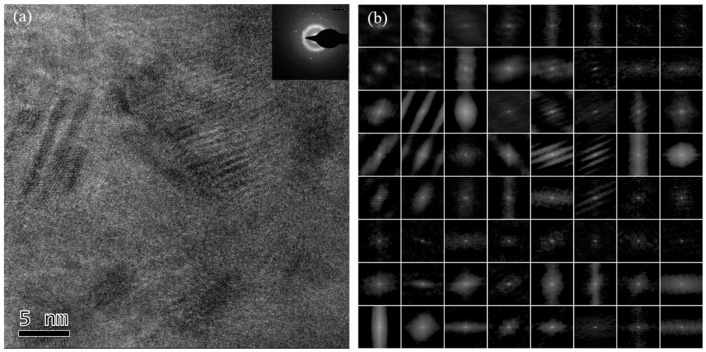
(**a**) HRTEM and (**b**) SAED images of the Cu3 sample.

High-resolution transmission electron microscopy (HRTEM) images and selective area electron diffraction (SAED) images of samples with the three different doping ratios are depicted in Figure 3, Figure 4 and Figure 5. The degree of order in the internal regional structure of the non-crystalline matrix in the microwires is quantitatively identified [15]. In this technique, the HRTEM images are divided into 64 regions, and each region undergoes processing by the auto-correlation function (ACF) in a digital micrograph to obtain ACF patterns. Based on a statistical standard, the entire region is split into two parts, where one part exhibits higher statistical degrees than the standard, and the other part has lower degrees. The statistical degree of the selective area electron diffraction (SAED) images is expressed as the auto-correlation function (ACF):

(1) Ψ=ζ/64×100%

where ζ represents the number of regions with higher statistical degrees than the auto-correlation standard, and k corresponds to the total number of changing patterns in the auto-correlation.

HRTEM images, including selective area electron diffraction (SAED) patterns and the ACF patterns of Cu0, Cu1, and Cu3 samples, are illustrated in Figure 3, Figure 4, and Figure 5, respectively.

For the Cu0 sample, the HRTEM image (Figure 3a) reveals a nearly uniform microcosmic structure distribution with no apparent ordered structure. The corresponding SAED pattern exhibited a diffraction halo with a blurred boundary, indicating a typical amorphous state. Analysis of multi-region electron diffraction imaging patterns (Figure 3b) revealed that most regions exhibited single-point amorphous diffraction patterns, and the statistical degree estimated by the auto-correlation function (ACF) is approximately 2%. Therefore, the amorphous degree of CoFeSiB microwires without Cu doping was notably high.

For the Cu1 sample, the high-resolution transmission image (Figure 4a) reveals uneven structure distribution in some areas, but no significant large-range ordered crystal structure was observed. The corresponding selective area electron diffraction (SAED) pattern exhibited a polycrystalline-like ring diffraction pattern, indicating a substantial number of organizations distributed orderly in the amorphous alloy substrate. The multi-region electron diffraction imaging pattern (Figure 4b) indicated that, in addition to the dot amorphous diffraction pattern, arrays of single crystal diffraction patterns also appear in some regions, estimated to be approximately 7% according to the auto-correlation function (ACF). Hence, nanocrystalline structure precipitation occurred in the CoFeSiB amorphous alloys with a 1% Cu doping ratio, wherein these nanocrystals had sizes smaller than 2 nm and were dispersed throughout the amorphous substrate.

For the Cu3 sample, the high-resolution transmission image (Figure 5a) exhibited a large range of striped ordered crystal structures with a structure scale larger than 5 nm. The SAED diffraction patterns contained single crystal-like diffraction bright spots and polycrystal-like ring patterns, indicating the presence of nanoscale single crystal regions within the alloys, distributed according to different lattice orientations. The multi-region electron diffraction imaging pattern (Figure 5b) comprised the dot amorphous diffraction pattern and arrays of single crystal diffraction patterns with different lattice orientations. The auto-correlation function (ACF) statistics estimate the alloy’s order to be 17%. Thus, when the Cu doping increased to 3%, the nanocrystal precipitation and the order of the alloys further increased.

**Figure 6:**
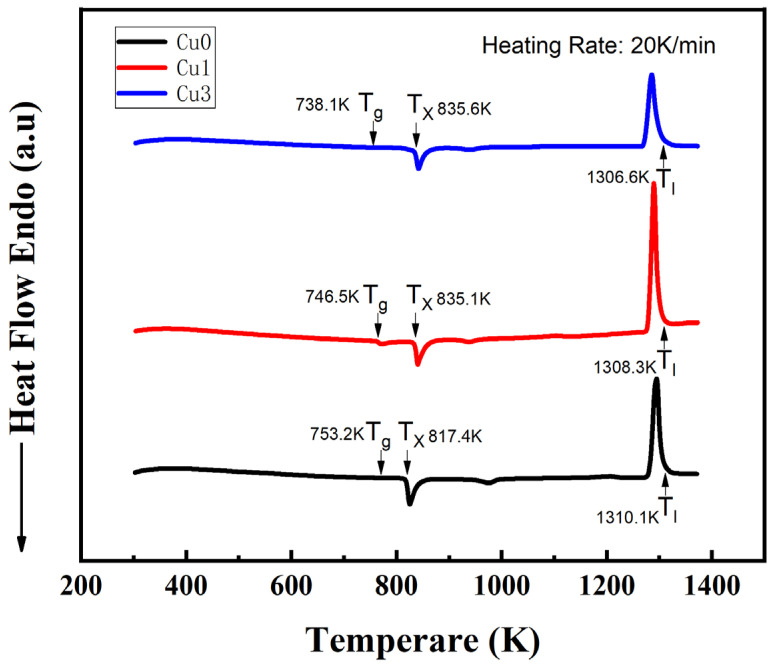
DSC curves of the amorphous microwires.

The TEM characterization results indicated that the doping of Cu atoms enhances the proportion of nanocrystalline-ordered components in the alloys. This improvement can be explained from the perspective of glass forming capacity (GFA), characterized using the reduced glass transition temperature system, T_rg_ = T_g_/T_l_, where T_g_ represents the glass conversion temperature, and T_l_ is the liquid phase point temperature of the alloys. A differential scanning calorimeter (DSC) was used to measure T_g_, T_l_, and the initial crystallization temperature T_x_ of the amorphous microwires (Figure 6).

The results are presented in Table 2. The T_rg_ values decreased with an increase in the Cu doping ratio. This indicates that the ability of the alloys to form an amorphous glass state gradually weakened with the doping of Cu atoms, making them more prone to crystal precipitation during the preparation process [16]. This result was consistent with the TEM findings.

Under the same preparation conditions, the CoFeSiB amorphous wire doped with Cu exhibited a positive correlation between the proportion of ordered structure precipitation and the amount of Cu elements. Moreover, Cu reduced the GFA parameter T_rg_ of the amorphous alloys under the same preparation conditions, promoting the formation of nanocrystalline crystals during the preparation process.

## 3. Soft Magnetic Properties and the Analysis

**Figure 7:**
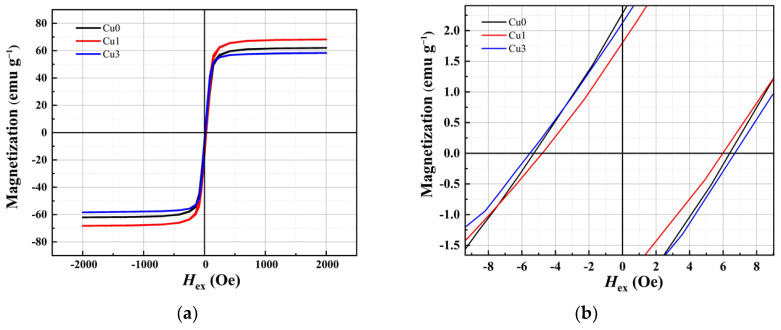
(**a**) M-H curves of the amorphous microwires; (**b**) the amplification near the origin.

The CoFeSiB amorphous wire hysteresis loop (M-H) was systematically tested using a Physical Property Measurement System (PPMS). The results, shown in Figure 7a, indicated that all samples exhibited the soft magnetic characteristic of low coercivity. The saturation magnetic induction strength of the Cu0 sample without Cu element doping is 62.1 emu/g. When the Cu doping amount increased to 1% in the Cu1 sample, the saturation magnetic induction strength increased to 68.3 emu/g. However, with a further increase in Cu doping to 3% in the Cu3 sample, the saturation magnetic induction strength decreased to 59.8 emu/g. Notably, the local amplification near the origin of the M-H curve (Figure 7b) indicated that the coercivity of Cu0 was 5.2 Oe without Cu element doping, whereas the value decreased to 4.8 Oe for Cu1 with 1% doping and increased to 5.7 Oe for Cu3 with 3% doping.

**Figure 8:**
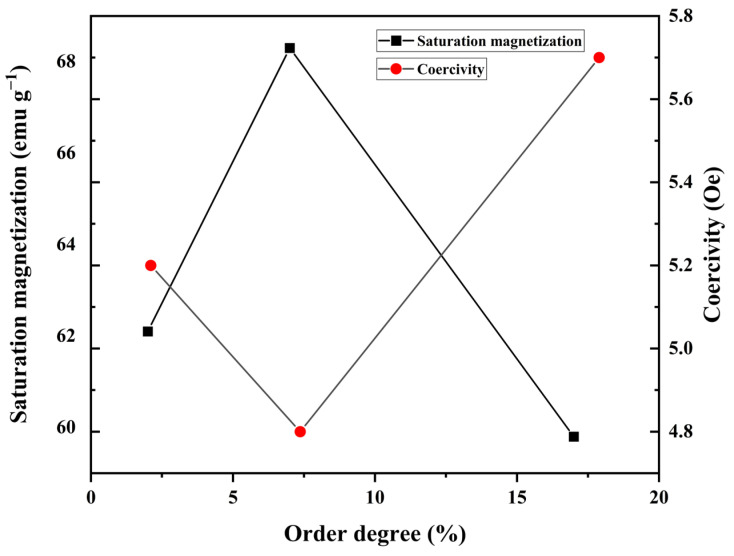
Dependence of the saturated magnetic sensing intensity (left) and the coercivity (right) on structure order degree.

Because the ratio of Cu doping determines the crystallization degree of the alloys, a relationship between the order degree of the alloys and their magnetic performance can be established (Figure 8). When the structure order was 2%, the CoFeSiB alloy exhibited the low coercivity and high saturation magnetic induction strength of an amorphous alloy. However, when the structure order increased to 7%, the alloy exhibited a decrease in coercivity and an increase in saturation magnetic induction intensity. This change may be attributed to the occurrence of a small number of nanocrystals in the alloy, with sizes much smaller than the magnetic exchange length. The considerably small average magnetic anisotropy energy of these nanoparticles did not hinder the movement and reversal of magnetic domain walls but enhanced the exchange between magnetic domains. When the structure order was 17%, the alloy exhibited an increase in coercivity and a decrease in saturated magnetic sensing intensity. Hence, when the scale and proportion of nanocrystalline structures increase, they hinder the movement of the magnetic domain walls, consequently reducing the soft magnetic performance. Therefore, the proportion and size of the nanocrystalline structures play a crucial role in determining the soft magnetic properties of the system.

## 4. Noise Test and Analysis

**Figure 9:**
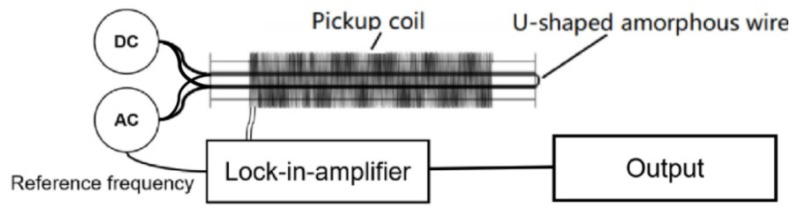
Basic schematic diagram of the probe structure and detection system of the magnetic field sensor.

To assess the impact of Cu-doped CoFeSiB amorphous wires, three Cu-doped wires were utilized as fundamental mode orthogonal fluxgate sensor probes to test the noise performance of the sensor. As depicted in Figure 9, the probe consisted of a U-type amorphous microwire as the magnetic core, with both the AC excitation current and DC bias current. The induced voltage signal at the base frequency, generated by the pickup coil, was analyzed and filtered by the lock-in amplifier with the excitation current frequency as the reference, and the output was recorded.

The essential parameters of the probe and excitation module are listed in Table 3. The induction coil length was 3 cm, with 400 turns, and the length of the U-type magnetic core is 4 cm. The AC excitation current was a sine waveform with an amplitude of 25 mA. The DC bias field was 50 mA, and the excitation frequency was 97 kHz.

**Figure 10:**
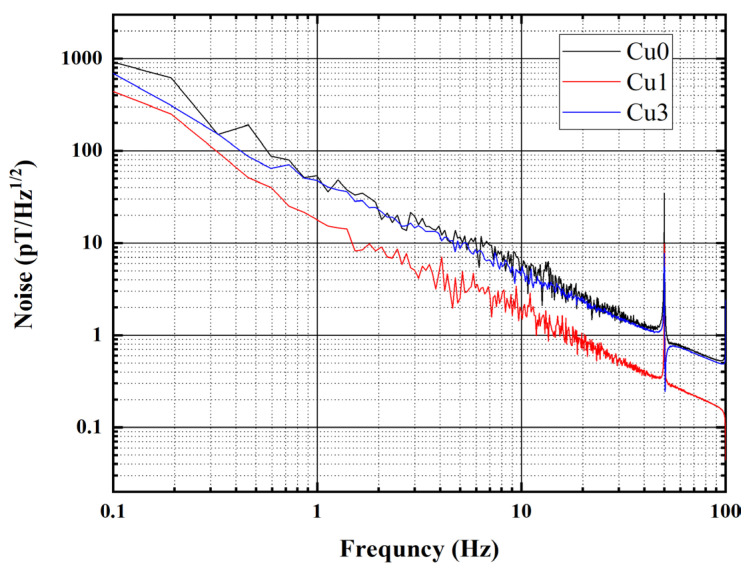
Power spectra noise of the fluxgate sensors.

The power spectra noise of these fluxgate sensors was measured, and the results are shown in Figure 10. When the Cu doping was 1%, the fluxgate sensor made of Cu1 amorphous microwire exhibits minimum noise at 1 Hz, approximately 20 pT/$\sqrt{\mathrm{Hz}}$. Without Cu doping, the noise of the Cu0 sensor is about 50 pT/$\sqrt{\mathrm{Hz}}$. When the Cu doping is 3%, the noise of the Cu3 fluxgate sensor is approximately 45 pT/$\sqrt{\mathrm{Hz}}$.

**Figure 11:**
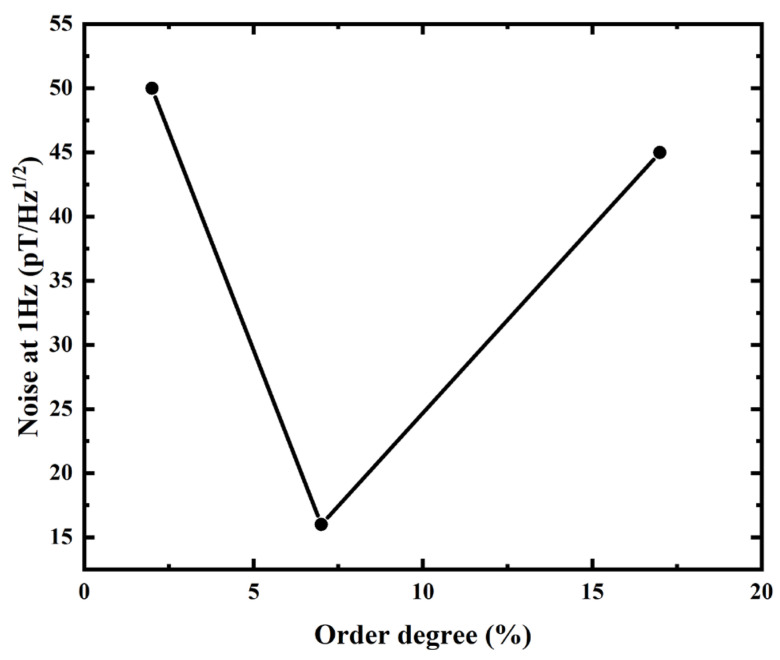
Dependence of the power spectra noise at 1 Hz on structure order degree.

The noise power spectral data at 1 Hz were utilized to characterize the noise level of the core materials (Figure 11). When the nanocrystalline increases, the noise initially decreases and then increases, with the lowest value obtained at the order degree of 7%. This trend is consistent with the change in the materials’ magnetic properties. The low noise performance is likely a result of the regulatory effect of the appeared nanocrystalline on the microscopic defects, such as residual stress and free volume voids, in the amorphous substrate. Additionally, the coupling effect between the small-sized nanocrystalline structure and the amorphous substrate contributes to the excellent soft magnetic properties of the alloys. This phenomenon is similar to that observed in Cr-doped CoFeSiB amorphous microwires.

The regulation of the nanocrystalline soft magnetic phase organization structure on the soft magnetic properties of amorphous alloys mainly stems from the coupling effect and defects reduction between the amorphous substrate and nanocrystalline. With an increase in the Cu doping ratio, the proportion and size of nanocrystalline in the alloys continuously increase. The alloys exhibit better sensor noise performances when there are smaller nanocrystalline sizes, leading to a decrease in the noise of a small amount of Cu-doped CoFeSiB amorphous microwire sensor. However, the proportions and sizes of nanocrystalline in the system increase with a further increase in the Cu doping ratio. At this stage, the oversized nanocrystalline phase induces mismatch stress with the substrate, resulting in a rise in the sensor noise again.

## 5. Conclusions

In this study, we investigated three types of Cu-doped CoFeSiB amorphous microwires prepared using MET. The growth of nanocrystalline structures was analyzed through TEM and DSC. Additionally, the magnetic performance of the amorphous microwires and the noise of the prepared fluxgate sensor were tested to establish the relationship between Cu-doped CoFeSiB amorphous microwires and their sensor noise performance. The research findings are summarized as follows:

(1) Cu doping significantly improved the crystallization degree of the CoFeSiB amorphous wire alloys. With an increase in the Cu doping ratio from 0 to 1% and then to 3%, the orders of the alloys increased from 2% to 7% and then to 17%, respectively. This enhancement indicates that Cu doping increased the content of ordered nanocrystalline structures in the CoFeSiB amorphous wire alloys. Furthermore, DSC analysis revealed that Cu doping reduced the glass formation capacity of the alloys.
(2) Cu doping exerted a notable influence on the soft magnetic properties of the amorphous wires. The low-doped sample with 1% Cu exhibited relatively low coercivity and higher saturated magnetic sensing intensity, which can be attributed to the interaction between nanocrystalline structures and magnetic domains.
(3) The sample with a 1% Cu doping ratio displayed the best noise performance, with the sensor showing a spectral noise at 1 Hz of less than 20 pT/$\sqrt{\mathrm{Hz}}$. The spectral noise at 1 Hz was 50 pT/$\sqrt{\mathrm{Hz}}$ and 45 pT/$\sqrt{\mathrm{Hz}}$ for the undoped and 3% Cu-doped samples, respectively. This behavior can be attributed to the coupling effect between the introduced mass-distributed nanocrystalline structures resulting from Cu doping and the amorphous substrate. This coupling effect contributed to lower coercivity and higher permeability, thus achieving the best soft magnetic properties with 1% Cu doping.

Therefore, to reduce the noise of the CoFeSiB amorphous microwire sensor, achieving excellent soft magnetic properties should be considered the primary goal. Doping the prepared CoFeSiB amorphous alloys with trace Cu elements is an effective method to directly adjust their properties. The conditions for this method are easier to control, ensuring the formation of stable amorphous microwires with uniform performance.

## Figures and Tables

**Table 1:** The nominal component proportions of microwires.

Sample	Co	Fe	Si	B	Cu
Cu0	68.15	4.35	12.25	15.25	0
Cu1	68.15	4.35	12.25	14.25	1
Cu3	68.15	4.35	12.25	12.25	3

**Table 2:** Results of the GFA analysis.

Sample	T_g_	T_x_	T_l_	T_rg_
Cu0	753.2 K	817.4 K	1310.1 K	0.57492
Cu1	746.5 K	835.1 K	1308.3 K	0.57059
Cu3	738.1 K	835.6 K	1306.6 K	0.5649

**Table 3:** Sensor probe and excitation module parameters.

	Parameters
Induction coil	3 cm long and 400 turns long
Magnetic core	4 cm U type
AC excitation current	Sine 25 mA
DC bias current	DC 50 mA
Pumping frequency	97 kHz

## Data Availability

The data presented in this study are available on request from the corresponding author.
